# The urgent need for disability studies among midlife adults

**DOI:** 10.1186/s40695-020-00057-w

**Published:** 2020-08-28

**Authors:** Carrie A. Karvonen-Gutierrez, Elsa S. Strotmeyer

**Affiliations:** Department of Epidemiology, Graduate School of Public Health, University of Pittsburgh, 130 N Bellefield Avenue, Suite 300, Pittsburgh, PA 15213 USA

**Keywords:** Women, Midlife, Middle age, Physical functioning, Functional limitations, Disability

## Abstract

Issues of poor physical functioning and disability are burdensome for midlife adults and evidence suggests that the prevalence of these conditions is increasing temporally. Physical functioning during the midlife period, however, may be highly amendable to intervention given the highly dynamic nature of functioning during this life stage. Thus, efforts to improve or forestall poor physical functioning and/or disability during midlife may not only improve the health status and quality of life for midlife adults but may have important ramifications on the health of these individuals who will become older adults in the future. This thematic series on women and disability includes contributions addressing issues of person, place and time with respect to disability in midlife and into late adulthood. The purpose of this commentary is to provide a summary overview of the major themes of the series and to offer insight into areas of most promise for intervention among midlife populations to improve physical functioning and prevent disability.

Issues of disability and limitations in physical functioning are commonly studied among older adults, but recent evidence suggests that the midlife period is a critical window for the onset of such limitations [1–3] and that limitations in functioning are common in midlife [1, 3, 4]. Disability and physical functioning limitations are measured in many different ways, guided by theoretical models of disablement [5], yet both concepts are reflective of one’s functional ability to carry out tasks necessary for independent living and engagement in society [6]. Data from the multi-site Study of Women’s Health Across the Nation (SWAN) found that the prevalence of physical functioning limitations increased from 19% among women ages 40–55 years to 50% among women 56–66 years of age [1, 2, 7]. Further, among SWAN women age 56–66 years of age, 19% reported severe to extreme mobility disability [8]. Not only do a high proportion of midlife adults experience states of disability, but nationally-representative panel surveys find that the burden of disability is expanding for midlife adults, unlike trends of stabilization or improvement among older adults (age 65–85 years) or the oldest old (85+ years) [9, 10]. Estimates suggest that over a third of 40–65 year-old Americans report limitations in functioning, but there have been larger increases in the burden among women than among men over the time period from 1997 to 2010 [11, 12].

Issues of midlife disability are compounded for under-represented minority (URM) populations, particularly Black adults, who have a higher prevalence of physical function limitations [2, 13–16]. Importantly, in the next several decades, the proportion of the older adult population that is non-Hispanic white is projected to drop from 77 to 55% [17, 18] with older URM increasing at a rate much higher than for the majority racial/ethnic population. Emerging views of disability also embrace life course approaches, particularly for URM populations, which consider risk accumulation across the lifespan as a pathway to disability including early adverse events, cumulative social and environmental exposures, and transgenerational transmission of risk and resilience [19–21].

Thus, issues of midlife onset, progression of disability, and racial/ethnic disparities in disability prevalence and trajectories are particularly concerning given the impact that the burgeoning issue of midlife disability has on productivity, health care costs, and risk for future adverse health events [22]. This special issue of Women’s Midlife Health provides a variety of contributions to exemplify the importance of disability as a midlife health issue and challenges us to consider outcomes and interventions once exclusively used in studies of older adults to expand our understanding of the health of midlife populations.

This special issue highlights disability among midlife women with respect to person, place, time and situation. A paper from the Gateway to Global Aging (Wang, Phillips and Lee) reports on gender differences in mid-life disability prevalence over a period of 10 years using harmonized data across 23 countries. While it is widely reported that women experience more disability than do age-matched men [3, 23] this analysis highlights important cross-country differences in disability prevalence and sex and gender disparities. These findings suggest the importance of considering both place and time in investigations of disability and consideration of the changing landscape of disability issues for women globally. Papers by Eagen et al. and Lange-Maia et al. highlight characteristics of individuals that must be considered when studying disability or planning for intervention programs. Findings from the National Fall Prevention Database (Eagen et al.) found that disability and fall rates were high among mid-life adults yet identified important sex differences in fall rates and important covariates. Those differences may be particularly relevant given findings in the Lange-Maia et al. paper, which demonstrated the importance of chronic conditions on poor functioning among midlife women. Thus, while the prevalence of chronic conditions is lower during mid-life than among older adults, this work suggests that individuals with early development of these conditions are at the greatest risk for poor functioning and disability. Identification of those individuals may offer opportunities for targeted intervention to maintain functioning and prevent disability. Issues of timing and type of interventions are considered in the commentary by Brown and Covinsky, which challenges readers to consider whether the midlife period is an opportune time to initiate activities to prevent or forestall the development of disabilities then and in late adulthood. These questions are not clear cut, and the evidence is evolving as we work as scientists, scholars, care providers, and policy makers to understand the best practices for individuals. In her personal commentary published in this special issue, Kessler shares some of the challenges she has encountered as a person with a disability in midlife and as a clinician treating those living with a disability, and how those challenges change and evolve as she continues to age. As Guest Editors of this Special Issue, we provide our own commentary below to highlight some of the most pressing needs for research in this area and to suggest areas that show most promise for intervention.

Once perceived broadly as a time of general good health, the midlife period is increasingly appreciated as an important life stage with respect to identifying individuals at risk of current or future adverse health outcomes. It is well-established that older adults with disability or limitations in functioning are at increased risk of hospitalization [24–26] and mortality [27–30]. However, data from the Health and Retirement Study (HRS), a nationally representative prospective cohort study, showed that this is also true among midlife adults. In that sample, individuals who developed a functional impairment during midlife had increased risk of hospitalizations and nursing home admission [31], thereby demonstrating that the burden of disabling states is clinically relevant among younger as well as older populations.

Issues of midlife disability and functioning are particularly relevant for women who may be multiply disadvantaged by lower socioeconomic status [32] and multiple caregiving responsibilities, both of which may impact physical and psychosocial stress. Findings from a Finnish study of working women demonstrate that physical functioning declines across the midlife period are more rapid for women of lower socioeconomic class as compared to women of higher socioeconomic class [33]. Similarly, findings from the United States National Health Interview Survey show that the temporal declines in physical functioning were greatest among individuals with the least education whereas levels of disability and functioning remained constant among midlife individuals with a college degree [34]. Understanding the ways in which socioeconomic status and caregiving responsibilities confer increased risk of disability, and differentially for women, is needed to develop strategies to effectively mitigate these risks.

Identifying factors that can be measured earlier in life that may predict risks for disability or adverse health factors is critically important so that we may understand which populations may benefit most from interventions. Not surprisingly, many of the factors that predict disability among midlife adults also predict disability among older adults, including frailty [35], high body mass index [36], and metabolic dysfunction [37]. A Canadian study explored correlates of activities of daily living (ADL) and instrumental activities of daily living and defined disability and social participation restriction among both midlife and older adults. In that study, chronic conditions including arthritis were major correlates of all disability constructs for midlife and older women whereas depression was particularly impactful on disability for midlife women [38]. The importance of depression as a correlate of disability was also observed in findings from the Michigan SWAN study. In that study, depressive symptomatology was the only condition consistently associated with all six domains of disability under the World Health Organization International Classification of Functioning model [8]. Other chronic conditions associated with selected domains of disability included knee osteoarthritis, hypertension and peripheral nerve impairment [8] – conditions also associated with poor physical functioning in SWAN [39, 40].

While several studies, including the Lange-Maia et al. paper in this issue, have been instrumental in bringing attention to the relationship between chronic conditions and disability among both younger and older populations, it is disheartening to see the overwhelming lack of research on these conditions across the spectrum of prevention research. A recent analysis by Vargas and colleagues [41] found that the number and percent of prevention research studies focused on leading risk factors for disability (measured using disability-adjusted life years) funded from 2012 to 2017 was disproportionate to the overall burden of those risk factors for disability. Further, the vast majority of studies examined by Vargas et al. [41] neglected to consider multiple risk factors for disability, despite well-documented evidence of the importance of multi-morbidity as a risk factor for disability [42, 43]. Thus, despite knowledge of the major risk factors for disability [44] and the potential public health impact by intervening on those risk factors, calculated as population attributable risks by Griffith et al. [38], scientific research on these topics for prevention of disability during midlife continues to be lacking. Concerns about the aging of the population, coupled with the increasing burden of disability among midlife populations, creates an urgent need for research to inform prevention and intervention strategies for issues of disability. Without this knowledge, care of individuals with disabling conditions will become economically unsustainable [45] and thus further create additional burden on individuals and family caregivers, thereby continuing to exacerbate the situation.

Thus, what is sorely needed is a scientific agenda leveraging what is known about correlates of disability among midlife populations to be translated into intervention programs to study whether poor functioning and disability can be prevented with early action. Evidence suggests that the time to act is during the midlife. Knowledge about the importance of midlife health and functioning informs questions of when and what type of interventions may be most efficacious in promoting health and preventing disability, both during the midlife and in late adulthood. Unlike in older adults, in whom physical functioning and disability tends to progressively worsen over time [46, 47], midlife declines are often reversible [2, 48]. In the HRS, 37% of midlife adults who developed a new ADL impairment were able to recover their independence within 2 years after the onset of that impairment and 28% remained independent for the next 10 years [48]. Similarly, SWAN evaluated the probability of worsening and improving physical functioning across five time points over a 10-year period. For midlife women in SWAN, the probability of improving physical functioning (either moving from some limitations to no limitations or from substantial limitations to some or no limitations) ranged from 11 to 30% over a two-year period whereas the probability of worsening was only 6–22% [2]. Both of these studies reinforce not only that midlife is the time when impairments in functioning begin, but that individuals during this life stage have the capacity for improvement. Thus, there is a need for greater attention and focus on the midlife period as it presents an opportune time for intervening early and initiating prevention strategies, before there are large and irreversible changes.

The interventions that hold the most promise for improving limitations and functioning are physical activity programs and healthy lifestyle programs. In older adults, sustained limitations in physical functioning lead to reduced physical activity and further declines in functioning [49]. The benefits of physical activity on functioning are not unique to older adults; in the Australian Longitudinal Study on Women’s Health, physical activity was a major predictor of optimal physical functioning for not only older women but also midlife and younger women [32]. In the SWAN study, an overall healthy lifestyle including a healthy diet, abstinence from smoking, and moderate levels of physical activity was associated with significantly better physical functioning, but this relationship was primarily driven by physical activity [50]. However, rates of physical activity are low among midlife women: only about one-half meet physical activity guidelines for aerobic activity and only one-quarter meet guidelines for muscle strengthening activities [51]. Interventions to improve physical activity in midlife adults have limited success [52], particularly when additionally paired with other health behavior interventions such as dietary change [53]. While the physical activity guidelines are the benchmark for optimal levels of activity among adults, the extremely high levels of sedentary behavior suggest that perhaps any activity is better than no activity. In fact, Spartano et al. [54] found that even very low levels (at least 5 min/day) of moderate to vigorous physical activity was associated with better performance on gait speed, chair stand and grip strength tests as compared to no activity [53]. However, physical activity interventions to improve physical functioning and avoid disability have been conducted almost exclusively in older adults [55]. Given the increasing burden of poor functioning and disability among midlife populations [22], particularly women and minority populations, coupled with the increasing risk of chronic conditions during this life stage [38, 43], it is important that physical activity be viewed as a highly actionable strategy for intervention. To do so, we must work to identify and address barriers to physical activity that may be particularly unique to midlife adults including time constraints, economic and safety constraints, and overall motivation. Understanding how to motivate midlife adults to engage in physical activity before they begin to experience functional changes is a particular challenge given that poor health is an often-cited factor triggering behavior change in older adults [56]. To fully leverage the promise of physical activity as a preventive factor for physical function limitations and disability, we must intervene at a point in time in which intervention can modify one’s risk of functional decline. Thus, it is imperative that future research be carried out to understand and identify key factors and features necessary to develop effective physical activity interventions and to test those interventions in midlife populations.

## Conclusion

In summary, a multitude of issues remain understudied in the origin and evolution of physical functioning decline and disability in midlife. With the implications of the impact on late-life disablement burden in women, midlife represents a time period of immense opportunity for maintenance of functional abilities and prevention of functional decline. Future research should target this key midlife period to understand how late-life disability may be mitigated.

## Data Availability

Not applicable.

## Abbreviations

SWAN: Study of Women’s Health Across the Nation
HRS: Health and Retirement Study
ADL: Activities of daily living
